# Novel Chloro-Substituted Salicylanilide Derivatives and Their β-Cyclodextrin Complexes: Synthesis, Characterization, and Antibacterial Activity

**DOI:** 10.3390/biomedicines10071740

**Published:** 2022-07-19

**Authors:** Ioana Maria Carmen Ienașcu, Adina Căta, Mariana Nela Ştefănuț, Iuliana Popescu, Gerlinde Rusu, Paula Sfîrloagă, Daniel Ursu, Cristina Moşoarcă, Anamaria Dabici, Corina Danciu, Delia Muntean, Raluca Pop

**Affiliations:** 1 National Institute of Research and Development for Electrochemistry and Condensed Matter, 144 Dr. A. P. Podeanu, 300569 Timişoara, Romania; ioanaienascu@incemc.ro (I.M.C.I.); mariana@incemc.ro (M.N.Ş.); paulasfirloaga@gmail.com (P.S.); danielursu@incemc.ro (D.U.); m.crristina@gmail.com (C.M.); anamaria.dabici@incemc.ro (A.D.); 2 Department of Pharmaceutical Sciences, Faculty of Pharmacy, “Vasile Goldiș” Western University of Arad, 86 Liviu Rebreanu, 310045 Arad, Romania; 3 Faculty of Agriculture, Banat’s University of Agricultural Sciences and Veterinary Medicine “King Michael I of Romania” from Timisoara, 119 Calea Aradului, 300645 Timișoara, Romania; 4 Faculty of Industrial Chemistry and Environmental Engineering, Politehnica University of Timișoara, 6 C. Telbisz, 300001 Timișoara, Romania; gerlinde.rusu@upt.ro; 5 Faculty of Pharmacy, “Victor Babeș” University of Medicine and Pharmacy, 2 Eftimie Murgu Square, 300041 Timișoara, Romania; corina.danciu@umft.ro (C.D.); muntean.delia@umft.ro (D.M.); pop.raluca@umft.ro (R.P.); 6 Research Centre for Pharmaco-Toxicological Evaluation, “Victor Babeș” University of Medicine and Pharmacy, 2 Eftimie Murgu Square, 300041 Timișoara, Romania; 7 Faculty of Medicine, “Victor Babeș” University of Medicine and Pharmacy, 2 Eftimie Murgu Square, 300041 Timișoara, Romania; 8 Multidisciplinary Research Center on Antimicrobial Resistance, “Victor Babeș” University of Medicine and Pharmacy, 2 Eftimie Murgu Square, 300041 Timișoara, Romania

**Keywords:** salicylanilide derivatives, inclusion complexes, antibacterial activity

## Abstract

The goal of this research was to design novel chloro-substituted salicylanilide derivatives and their β-cyclodextrin complexes in order to obtain efficient antibacterial compounds and to demonstrate the beneficial role of complexation on the efficiency of these compounds. Thus, salicylanilide derivatives, esters, and hydrazides were obtained by microwave-assisted synthesis and their structure proven based on FTIR and NMR spectra. In order to improve water solubility, chemical and physical stability, and drug distribution through biological membranes, the inclusion complexes of the ethyl esters in β-cyclodextrin were also obtained using kneading. Inclusion-complex characterization was accomplished by modern analytical methods, X-ray diffraction, SEM, TGA, FTIR, and UV-vis spectroscopy. The newly synthesized compounds were tested against some Gram-positive and Gram-negative bacteria. Antimicrobial tests revealed good activity on Gram-positive bacteria and no inhibition against Gram-negative strains. The MIC and MBC values for compounds derived from N-(2-chlorophenyl)-2-hydroxybenzamide were 0.125–1.0 mg/mL. N-(4-chlorophenyl)-2-hydroxybenzamide derivatives were found to be less active. The inclusion complexes generally behaved similarly to the guest compounds, and antibacterial activity was not been altered by complexation.

## 1. Introduction

Salicylanilides have been extensively studied in medicinal chemistry due to the varied biological activities related to their remarkable in vitro antibacterial, antimicrobial, antifungal, and antimycobacterial effects [1,2,3]. Novel salicylanilides were also been tested as inhibitors of epidermal growth factor receptor (EGFR) protein tyrosine kinases, the overexpression of this enzyme being associated with oncogenic activity [4].

The biological activity of salicylanilides may be influenced by their hydrophobicity. Even if a phenolic group seems to be essential for the antimicrobial activity, it could confer irritative properties, being also responsible for uncoupling activity. Thus, temporary blocking of phenolic groups by transformation of salicylanilides in esters could exert high activity, improved bioavailability, easier penetration of the membranes, and lower toxicity. It is not known exactly if salicylanilide esters behave as prodrugs with absent or insignificant in vitro biological activity or if they can be considered novel structural entities with their own specific activity. The first supposition is based on the fact that salicylanilide esters are hydrolyzed in plasma, the ester form being only a transitory transport form until transformation into salicylanilides with a free phenolic group [5]. The second supposition relies on studies [6,7] revealing that many salicylanilide esters, when compared with parent phenolic molecules, displayed higher antimicrobial activity. A combination of these two hypotheses should not be disregarded either.

On the other hand, salicylamidoacetic acid hydrazide shows superior anti-inflammatory and analgesic activity to salicylamide itself and lower ulcerogenic activity [8]. Heterocyclic compounds 2-(N-piperidinyl)acetic acid hydrazide and 2-methyl-3-(N-piperidinyl) propionic acid hydrazide show high antimicrobial and spasmolytic activity along with low toxicity [9]. Isoniazid (isonicotinic acid hydrazide, INH) is used as a first-line drug in the treatment and prophylaxis of tuberculosis, although it has shown multiple side effects (i.e., hepatotoxicity) [10]. The antimycobacterial activity of isoniazid was proven in 1952, and immediately, INH-resistant *Mycobacterium tuberculosis* strains were reported [11]. As such, along with the frightening increase in antimicrobial resistance, it seems that the combination of a salicylamide moiety and a hydrazide group could be a proper way of designing new antimicrobial agents to combat resistant pathogens.

Salicylanilide hydrophobicity and irritative effects could also be countered using cyclodextrins. Cyclodextrin inclusion complexes are broadly used in food, cosmetics and especially the pharmaceutical industry, because their drug-delivery systems are distinguished by increased biodisponibility, solubility, stability, relatively low toxicity (high therapeutic index), improved pharmacokinetic properties, and low price [12,13,14,15]. Complex formation is based on hydrophobic forces and the size of guest molecules and their properties, elements that are responsible for the complexes’ stability. The process of partial or total encapsulation of a guest molecule occurs when a hydrophobic molecule or just a hydrophobic fragment of a polar molecule is integrated in the cyclodextrin (CD) cavity, causing a shift of water molecules from the cavity and as a result increasing the aqueous solubility of the sample [16].

However, few researchers have studied the interaction between salicylanilide and β-cyclodextrin. Such inclusion complexes have been investigated by UV and fluorescence techniques that validated the insertion of the -NH-CO-C_6_H_5_ aromatic side chain of salicylanilide inside the cyclodextrin cavity. The inclusion phenomenon was an exergonic and spontaneous process [17]. In a recent work [18], we also demonstrated the liquid phase formation of an ethyl [2-(2-bromophenylcarbamoyl)phenoxy]acetate/β-cyclodextrin complex, 1:1 stoichiometry and the apparent formation constant (K = 1211 ± 111.83 L/mol) being established using absorbance measurements and the Benesi–Hildebrand equation. The inclusion compound’s geometry was established using molecular modeling, which, alongside ^1^H-NMR, proved that the ethyl ester is included with the benzamide moiety inside the β-CD cavity.

The aim of this research was to obtain and characterize novel chloro-substituted salicylanilide derivatives and their β-cyclodextrin complexes, and to evaluate the antibacterial activity of the compounds in this class.

## 2. Materials and methods

### 2.1. Chemicals

Reagents: 2-chloroaniline, 4-chloroaniline, phosphorus trichloride (Acros Organics, Geel, Belgium, for synthesis); salicylic acid, ethyl chloroacetate, methyl chloroacetate, hydrazine monohydrate, β-cyclodextrin (Sigma-Aldrich, St. Louis, MO, USA, for synthesis) and solvents: chlorobenzene, absolute ethanol, dimethylformamide, dimethyl sulfoxide (DMSO), 2-butanone (Merck, Darmstadt, Germany, analytical purity), were used as purchased.

### 2.2. Apparatus

Microwave-assisted syntheses was carried out using a Speed Wave MWS–2 microwave oven (Berghof, Germany). Melting points (uncorrected) were established using Stuart SMP30 Melting Point apparatus (Bibby Scientific Limited, Stone, UK). FTIR spectra (KBr pellet, ν_max_ in cm^−1^) were acquired on a Vertex 70 FTIR apparatus (Bruker, Ettlingen, Germany). ^1^H and ^13^C-NMR spectra (DMSO-d_6_, 400 MHz) were recorded on an Avance DRX 400 instrument (Bruker, Billerica, MA, USA). Chemical shifts (δ values in ppm) are expressed aligned with tetramethylsilane (TMS). Coupling constants (*J*) are given in Hz. X-ray diffraction (XRD) measurements were performed using an X’Pert PRO MPD apparatus (PANalytical, Almelo, The Netherlands). Scanning electron microscopy (SEM) analysis was performed using an Inspect S microscope (FEI, Eindhoven, Holland). Thermogravimetric analysis (TGA) was carried out on a TG 209F1 Libra (Netzsch, Selb, Germany), and UV-Vis measurements were carried out on a UV-Vis-NIR Lambda 950 (PerkinElmer, Waltham, MA, USA).

### 2.3. Microwave-Assisted Syntheses

*Ethyl/Methyl esters* (EE2, EE4, ME2, ME4). A mixture of an appropriate anilide (0.0025 mol), chloro-acetic acid alkyl ester (0.0025 mol), potassium carbonate (0.0025 mol), and 2-butanone (10 mL) was poured into a special Teflon tube and irradiated in a microwave oven at 500 W, 150 °C, for 10 min. After cooling, the obtained solid products were filtered, water-washed, dried out, and re-crystallized from ethanol.

*Hydrazides* (HD2, HD4). The appropriate ethyl ester (0.0025 mol), hydrazine hydrate (0.0025 mol), and absolute ethanol (10 mL) were mixed together, poured into a special Teflon tube, and irradiated in a microwave oven at 500 W, 150 °C, for 6 min. After cooling, the precipitate was filtered, water-washed, dried, and re-crystallized from ethanol.

### 2.4. Obtaining the Inclusion Complexes

The inclusion complexes (CEE2, CEE4) of ethyl 2-(2-((2-chlorophenyl) carbamoyl)phenoxy)acetate (EE2) and ethyl 2-(2-((4-chlorophenyl)carbamoyl)phenoxy) acetate (EE4) in β-cyclodextrin (CD) were obtained using the kneading method. The two components (1:1 molar ratio) were ground in an agate mortar for 60 min, with ethanol added to obtain a paste. The obtained product was held in a heating chamber at 60 °C for 4 days and stored in a desiccator.

### 2.5. Obtaining the Physical Mixtures

The physical mixtures (PMEE2, PMEE4) of β-cyclodextrin and ethyl 2-(2-((2-chlorophenyl)carbamoyl)phenoxy)acetate and ethyl 2-(2-((4-chlorophenyl) carbamoyl)phenoxy)acetate were prepared. First, the β-cyclodextrin and ethyl ester were individually mixed in a mortar. Then, both components, at a molar ratio of 1:1, were mixed together to form a unitary mixture [19].

### 2.6. Antibacterial Activity Evaluation

#### 2.6.1. Test Compounds

All newly obtained compounds, four esters, two hydrazides, and two inclusion complexes were evaluated for their antimicrobial properties. Five serial dilutions in DMSO of test compounds, from 10 to 0.625 mg/mL, were obtained and used for in vitro tests.

#### 2.6.2. Bacterial Strains

Five bacterial strains, Gram-positive and Gram-negative bacteria, were evaluated for their susceptibility to the test compounds. The selected strains, *Streptococcus mutans* ATCC 35668, *Streptococcus pyogenes* ATCC 19615, *Staphylococcus aureus* ATCC 25923, *Escherichia coli* ATCC 25922, and *Pseudomonas aeruginosa* (ATCC 27853, Microbiologics, France) were reference strains specific to human bacteria infections. The strains were reconstituted by inoculation on Columbia agar +5% sheep blood (bioMérieux, France), then the plates were incubated for 24 h at 37 °C.

#### 2.6.3. Determination of Minimum Inhibitory Concentration (MIC) and Minimum Bactericidal Concentration (MBC)

The antibacterial properties of the tested compounds were evaluated by dilution method adapted to the requirements of the European Committee on Antimicrobial Susceptibility Testing (EUCAST), Clinical Laboratory and Standard Institute (CLSI), and other studies [20,21,22,23,24].

From each strain, standardized suspensions were prepared to a concentration of 0.5 McFarland and this inoculum was adjusted by dilution at 10^5^ CFU/mL. Then, 100 µL of each test compound dilution, 400 µL Mueller-Hinton broth (bioMérieux, Marcy-l’Etoile, France), supplemented with horse blood and β-NAD for streptococci, and 500 µL bacterial suspension were added to the test tubes and incubated for 24 h at 37 °C. Thus, the final dilutions of the test compounds were considered ten times lower than the initial serial dilutions. The higher dilution with no observable growth was considered the minimum inhibitory concentration (MIC). To determine the minimum bactericidal concentration (MBC), which is the higher dilution that killed 99.9% of the bacteria, 1 µL from the obtained suspension with no visible growth was inoculated on Columbia agar with sheep blood (bioMérieux, Marcy-l’Etoile, France) and incubated for 24 h at 37 °C.

The negative control consisted of 100 µL of DMSO, 400 µL broth, and 500 µL bacterial suspension.

## 3. Results and Discussion

### 3.1. Synthesis of the Chloro-Substituted Salicylanilide Derivatives

The anilides used as raw materials for ester synthesis, N-(2-chlorophenyl)-2-hydroxybenzamide and N-(4-chlorophenyl)-2-hydroxybenzamide, were prepared and purified according to [25].

Esters and hydrazides were obtained by irradiation in a microwave field, an eco-friendly method with higher yields and solvent saving compared to the conventional heating method [26].

**Scheme 1:**
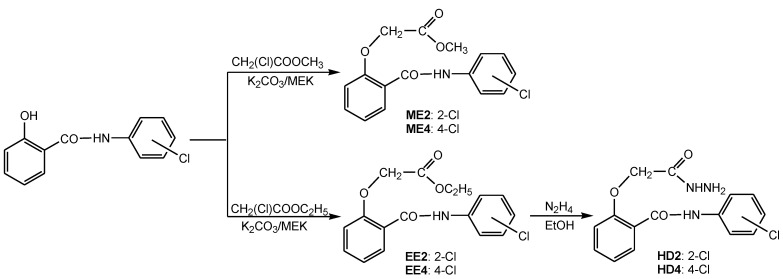
Synthetic pathway for salicylanilide derivatives.

The synthesized compounds (Scheme 1) were white or gray crystalline substances, obtained with yields between 65–97%.

### 3.2. Characterization of the Chloro-Substituted Salicylanilide Derivatives

The identity of the novel synthesized chloro-substituted salicylanilide derivatives was confirmed using FTIR and NMR analyses.

***Ethyl 2-(2-((2-chlorophenyl)carbamoyl)phenoxy)acetate (EE2).*Yield** (%): 84, **m.p.** (°C): 71–72. **IR** ν(cm^−1^) KBr pellet: 3327m (νNH free sec. amide); 3077s (νCH aromatic); 1741m (νC=O ester); 1677m (νC=O amide); 1592m (Sk “1600”); 1535m (γNH + νCN sec. amide); 1485m, 1455m, 1438m (Sk “1500”); 1391m (σCH_3_ aliphatic); 1378m (σCH aromatic); 1311i (ν^as^COC aromatic); 1247m (γCN + νNH sec. amide); 1161m (ν^as^COC aliphatic); 1100m (ν^s^COC aromatic); 754m (Sk “700”, aromatic nucleus 1,2-disubstituted). **^1^H-NMR** [δ(ppm)]: 1.16 (t, 3H, -CH_2_CH_3_, *J* = 8.0); 4.17 (q, 2H, -CH_2_CH_3_, *J* = 8.0); 5.18 (s, 2H, Ar-OCH_2_-); 7.20 (t, 2H, H_3_,H_10_, *J* = 8.0); 7.25 (d, 1H, H_5_, *J* = 8.0); 7.40 (t, 1H, H_11_, *J* = 8.0); 7.53–7.60 (m, 2H, H_4_, H_9_); 8.08 (d, 1H, H_12_, *J* = 8.0); 8.31 (d, 1H, H_2_, *J* = 8.0); 10.42 (s, 1H, -NH-). **^13^C-NMR** [δ(ppm)]: 13.90 (-CH_2_CH_3_); 61.14 (-CH_2_CH_3_); 65.70 (Ar-OCH_2_-); 113.65 (C_5_); 121.65 (C_1_); 122.05 (C_3_); 123.48 (C_12_); 124.24 (C_10_); 125.55 (C_8_); 127.74 (C_11_); 129.37 (C_2_); 131.61 (C_9_); 133.71 (C_4_); 135.10 (C_7_); 155.59 (C_6_); 162.78 (Ar-CO); 168.37 (-COO-).

***Ethyl 2-(2-((4-chlorophenyl)carbamoyl)phenoxy)acetate (EE4).*Yield** (%): 79, **m.p.** (°C): 157–158. **IR** ν(cm^−1^) KBr pellet: 3323m (νNH free sec. amide); 3101s (νCH aromatic); 1749m (νC=O ester); 1662m (νC=O amidă); 1593m (Sk “1600”); 1533m (γNH + νCN sec. amide); 1487m, 1436m (Sk “1500”); 1392m (σCH_3_ aliphatic); 1361m (σCH aromatic); 1317i (ν^as^COC aromatic); 1226m (γCN + νNH sec. amide); 1157m (ν^as^COC aliphatic); 1093m (ν^s^COC aromatic); 842i, 756i (Sk “700”, aromatic nuclei 1,2- and 1,4-disubstituted). **^1^H-NMR** [δ(ppm)]: 1.23 (t, 3H, -CH_2_CH_3_, *J* = 8.0); 4.24 (q, 2H, -CH_2_CH_3_, *J* = 8.0); 5.00 (s, 2H, Ar-OCH_2_-); 7.16 (t, 1H, H_3_, *J* = 8.0); 7.19 (d, 1H, H_5_, *J* = 8.0); 7.43 (d, 2H, H_11_, H_9_, *J* = 8.0); 7.54 (t, 1H, H_4_, *J* = 8.0); 7.86 (d, 2H, H_12_, H_8_, *J* = 8.0); 7.90 (d, 1H, H_2_, *J* = 8.0); 10.50 (s, 1H, -NH-). **^13^C-NMR** [δ(ppm)]: 14.02 (-CH_2_CH_3_); 61.20 (-CH_2_CH_3_); 65.78 (Ar-OCH_2_-); 113.57 (C_5_); 121.44 (C_12_,C_8_); 121.72 (C_1_); 123.00 (C_3_); 127.30 (C_2_); 128.68 (C_11_,C_9_); 130.86 (C_10_); 133.02 (C_4_); 137.87 (C_7_); 155.07(C_6_); 163.39 (Ar-CO); 168.88 (-COO-).

***Methyl 2-(2-((2-chlorophenyl)carbamoyl)phenoxy)acetate (ME2).*Yield** (%): 65, **m.p.** (°C): 85–87. **IR** ν(cm^−1^) KBr pellet: 3345m (νNH free sec. amide); 3082s (νCH aromatic); 1751i (νC=O ester); 1678i (νC=O amide); 1596i(Sk “1600”); 1541i (γNH + νCN sec. amide); 1486m, 1457i, 1437m (Sk “1500”, σ_as_CH_3_ ester); 1377m (σCH aromatic); 1308m (ν^as^COC aromatic); 1240m (γCN + νNH sec. amide); 1200i (ν^as^COC aliphatic); 1067m (ν^s^COC aromatic); 747i (Sk“700”, aromatic nucleus 1,2-disubstituted). **^1^H-NMR** [δ(ppm)]: 3.71 (s, 3H, -CH_3_); 5.20 (s, 2H, Ar-OCH_2_); 7.19–7.25 (m, 3H, H_3_, H_5_, H_10_); 7.39 (t, 1H, H_11_, *J* = 8.0); 7.56 (d, 1H, H_9_); 7.58 (t, 2H, H_4_); 8.06 (d, 1H, H_12_, *J* = 8.0); 8.29 (d, 1H, H_2_, *J* = 8.0); 10.40 (s, 1H, -NH-). **^13^C-NMR** [δ(ppm)]: 52.21 (-CH_3_); 65.57 (Ar-OCH_2_-); 113.62 (C_5_); 121.64 (C_1_); 122.06 (C_3_); 123.56 (C_12_); 124.32 (C_10_); 125.63 (C_8_); 127.77 (C_11_); 129.40 (C_2_); 131.60 (C_9_); 133.73 (C_4_); 135.08 (C_7_); 155.55 (C_6_); 162.80 (Ar-CO); 168.92 (-COO-).

***Methyl 2-(2-((4-chlorophenyl)carbamoyl)phenoxy)acetate (ME4).*Yield** (%): 69, **m.p.** (°C): 142–143. **IR** ν(cm^−1^) KBr pellet: 3329m (νNH free sec. amide); 3047s (νCH aromatic); 1753i (νC=O ester); 1654i (νC=O amide); 1599i (Sk “1600”); 1541i (γNH + νCN sec. amide); 1487m, 1494i, 1452m (Sk “1500”, σ_as_CH_3_ ester); 1317m (ν^as^COC aromatic); 1228m (γCN + νNH sec. amide); 1164i (ν^as^COC aliphatic); 1064m (ν^s^COC aromatic); 830i, 752i (Sk “700”, aromatic nuclei 1,2- and 1,4-disubstituted). **^1^H-NMR** [δ(ppm)]: 3.78 (s, 3H, -CH_3_); 5.02 (s, 2H, Ar-OCH_2_); 7.16 (t, 1H, H_3_, *J* = 8.0); 7.19 (d, 1H, H_5_, *J* = 8.0); 7.43 (d, 2H, H_11_, H_9_, *J* = 8.0); 7.54 (t, 1H, H_4_, *J* = 8.0); 7.86 (d, 2H, H_12_, H_8_, *J* = 8.0); 7.90 (d, 1H, H_2_, *J* = 8.0); 10.50 (s, 1H, -NH-). **^13^C-NMR** [δ(ppm)]: 52.26 (-CH_3_); 65.69 (Ar-OCH_2_-); 113.53 (C_5_); 121.41 (C_12_,C_8_); 121.72 (C_1_); 122.93 (C_3_); 127.29 (C_2_); 128.68 (C_11_,C_9_); 130.87 (C_10_); 133.02 (C_4_); 137.85 (C_7_); 155.03 (C_6_); 163.34 (Ar-CO); 169.32 (-COO-).

***N-(2-Chloro-phenyl)-2-hydrazinocarbonylmethoxy-benzamide (HD2).*Yield** (%): 97, **m.p.** (°C): 164–166. **IR** ν(cm^−1^) KBr pellet: 3313m (νNH assoc. sec. amide, νNH + νNH_2_ hydrazide); 1657m (νC=O hydrazide, νC=O amidă); 1591m (σNH_2_ hydrazide); 1546m (Sk “1600”, γNH + νCN sec. amide, νCN hydrazide); 1487m, 1455m (Sk “1500”); 1261s (γCN + νNH sec. amide); 1212m (ν^as^COC aromatic); 1032m (ν^s^COC aromatic); 968m (σCH aromatic); 753m (Sk “700”, aromatic nucleus 1,2-disubstituted). **^1^H-NMR** [δ(ppm)]: 4.55 (s, 2H, -NH_2_); 4.89 (s, 2H, Ar-OCH_2_-); 7.16 (d, 1H, H_5_);7.18 (t, 1H, H_10_);7.21 (t, 1H, H_3_); 7.40 (t, 1H, H_11_, *J* = 8.0); 7.56 (m, 2H, H_4_, H_9_); 8.03 (d_sc_, 1H, H_12_, *J* = 8.0); 8.25 (d, 1H, H_2_, *J* = 8.0); 9.58 (s, 1H, -NH-NH_2_); 10.56 (s, 1H, -NH-Ar). **^13^C-NMR** [δ(ppm)]: 66.92 (Ar-OCH_2_-); 113.59 (C_5_); 121.97 (C_1_); 122.53 (C_3_); 124.17 (C_12_); 124.94 (C_10_); 125.71 (C_8_); 127.69 (C_11_); 129.45 (C_2_); 131.49 (C_9_); 133.43 (C_4_); 135.40 (C_7_); 155.89 (C_6_); 163.29 (Ar-CONH-); 166.58 (-CO-NH-NH_2_).

***N-(4-Chloro-phenyl)-2-hydrazinocarbonylmethoxy-benzamide (HD4).*Yield** (%): 95, **m.p.** (°C): 192–195. **IR** ν(cm^−1^) KBr pellet: 3339i (νNH assoc. sec. amide); 3276i (νNH + νNH_2_ hydrazide); 1638m (νC=O hydrazide, νC=O amide); 1599m (σNH_2_ hydrazide); 1546m (Sk “1600”, γNH + νCN sec. amide, νCN hydrazide); 1493m, 1444m (Sk “1500”); 1234m (ν^as^COC aromatic); 1046m (ν^s^COC aromatic); 988m (σCH aromatic); 830i, 753i (Sk “700”, aromatic nuclei 1,2- and 1,4-disubstituted). **^1^H-NMR** [δ(ppm)]: 4.59 (s, 2H, -NH_2_); 4.82 (s, 2H, Ar-OCH_2_-); 7.13–7.17 (m, 2H, H_5_, H_3_); 7.42 (d, 2H, H_11_, H_9_, *J* = 8.0); 7.53 (t, 1H, H_4_); 7.82 (d, 1H, H_2_, *J* = 8.0); 7.90 (d, 2H, H_12_, H_8_, *J* = 8.0); 9.56 (s, 1H, -NH-NH_2_); 10.88 (s, 1H, -NH-Ar). **^13^C-NMR** [δ(ppm)]: 66.79 (Ar-OCH_2_-); 113.84 (C_5_); 121.37 (C_12_,C_8_); 121.87 (C_1_); 124.28 (C_3_); 127.25 (C_2_); 128.70 (C_11_,C_9_); 130.77 (C_10_); 132.83 (C_4_); 138.09 (C_7_); 155.28(C_6_); 164.04 (Ar-CONH-); 167.34 (-CO-NH-NH_2_).

IR spectra of the esters showed the formation of ether bonds by bands in the ranges 1300–1320 cm^−1^ (ν_as_COC aromatic) and 1150–1210 cm^−1^ (ν_as_COC aliphatic). Ester carbonyl groups (νC=O) appeared between 1740 and 1760 cm^−1^. These bands were not present in the IR spectra of the hydrazides, attesting the transformation of the esters into hydrazides. The bands associated with the vibrations of the amidic and hydrazidic groups appeared amid 3270–3350 cm^−1^ (νNH) and 1630–1680 cm^−1^ (νC=O).

**Scheme 2:**
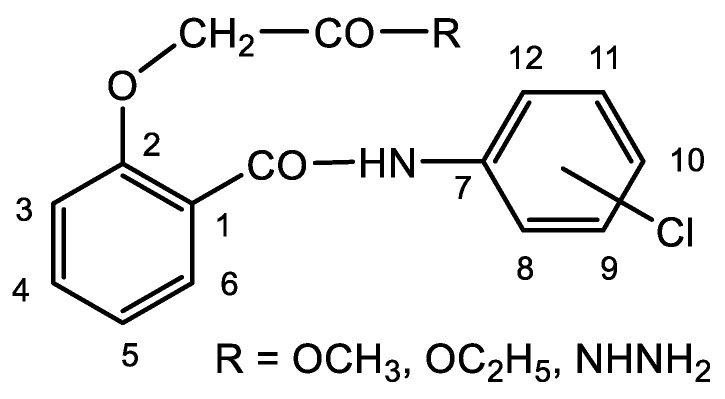
The numbering of aromatic nuclei.

For NMR data explanation, in Scheme 2, the numbering of the aromatic nuclei is shown.

The ^1^H-NMR signals of methyl and ethyl groups from esters appeared between 1.15 and 4.25 ppm, those of the amide group between 10.40 and 10.90 ppm, and those of the hydrazide group between 9.55 and 9.60 ppm.

The ^13^C-NMR signals of the hydrazide and amide carbons are seen at 162–168 ppm and those for aromatic carbons at 113–156 ppm.

### 3.3. Characterization of the Inclusion Complexes

**Scheme 3:**
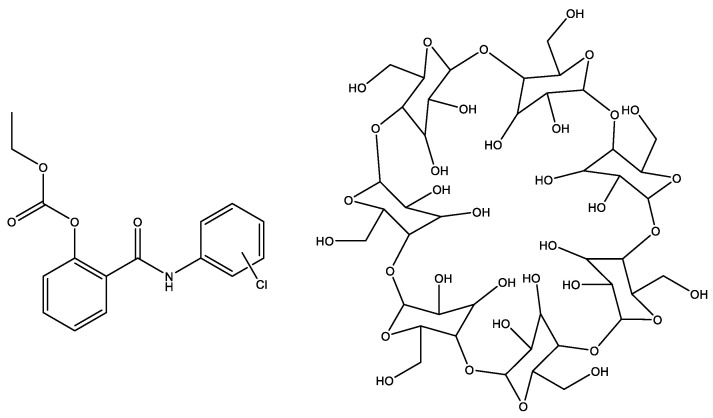
Structures of the two components of the ethyl ester–β-cyclodextrin complexes.

The molecules of ethyl esters (Scheme 3, left) are apolar and can be included in the central cavity of β-cyclodextrin (Scheme 3, right), also apolar.

For a better understanding of the inclusion complex formation, alongside the complexes, physical mixtures containing the two components of the complex were also prepared and analyzed.

Inclusion complex characterization was accomplished based on XRD, SEM, TGA, FTIR, and UV-vis spectroscopy.

**Figure 1:**
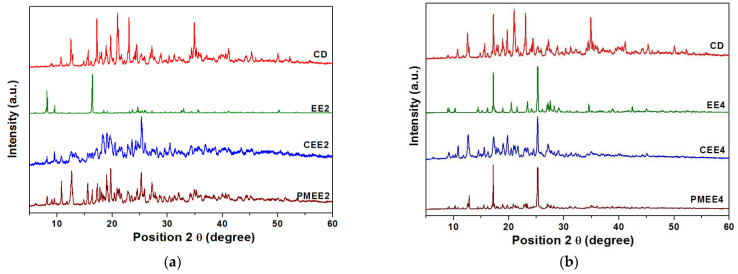
XRD spectra of β-cyclodextrin, ethyl ester, inclusion complex, physical mixture: (**a**) ethyl 2-(2-((2-chlorophenyl)carbamoyl)phenoxy)acetate series; (**b**) ethyl 2-(2-((4-chlorophenyl) carbamoyl)phenoxy)acetate series.

Figure 1 presents the X-ray diffraction spectra of the ethyl esters, β-cyclodextrin, and their physical mixtures and inclusion complexes. Diffractograms were recorded to study the change in crystal morphology from the parent crystalline molecules.

X-ray diffraction indicated that in the case of the complexes (CEE2, CEE4), there was a decrease in the degree of crystallinity compared to the guest substance (EE2 and EE4) due to the amorphization phenomenon, respectively, a change in the position and intensity of the diffraction peaks. These modifications attested the formation of a new solid state and demonstrated the existence of interactions between the esters and the cyclodextrin.

**Figure 2:**
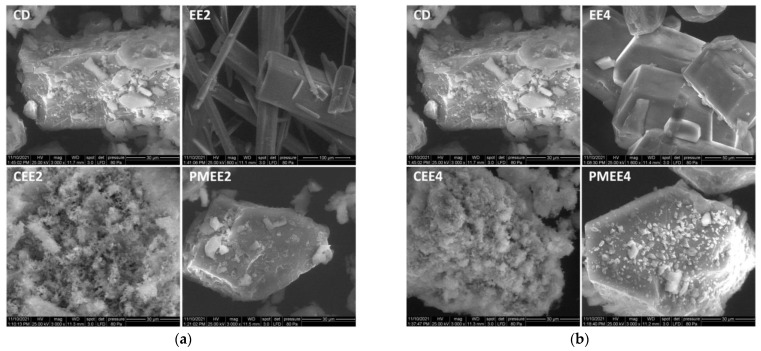
SEM images of β-cyclodextrin, ethyl ester, inclusion complex, physical mixture: (**a**) ethyl 2-(2-((2-chlorophenyl)carbamoyl)phenoxy)acetate series; (**b**) ethyl 2-(2-((4-chlorophenyl) carbamoyl)phenoxy)acetate series.

In Figure 2, SEM images of the ethyl esters, β-cyclodextrin, their physical mixtures, and inclusion complexes are shown. The SEM images were recorded in order to evaluate the surface morphology and dimension of the crystals. For ethyl esters, the obtained crystals were rod-shaped (EE2) or prisms (EE4). As for the inclusion complexes, amorphous aggregates with asymmetrical shapes were observed. The particles were less agglomerated, and their dimensions were in the range of hundreds of nanometers. Regarding the obtained physical mixtures (PMEE2 and PMEE4), there were no major difference between these and the CD sample. The crystals were more agglomerated.

**Figure 3:**
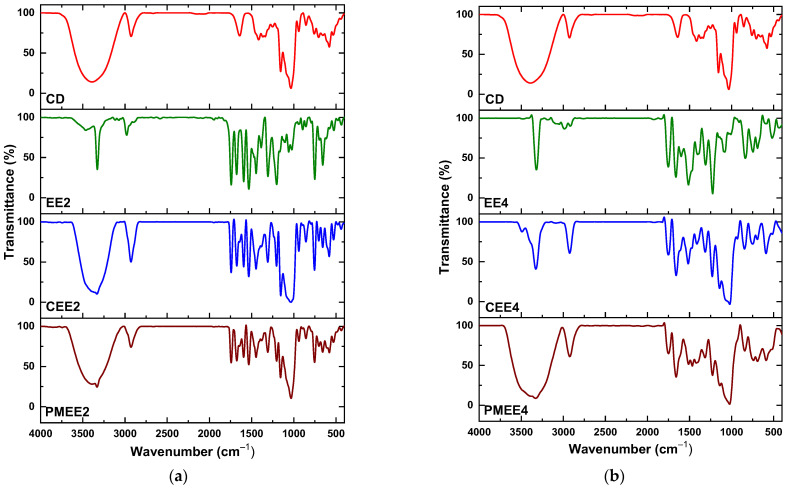
FTIR spectra of β-cyclodextrin, ethyl ester, inclusion complex, physical mixture: (**a**) ethyl 2(2-((2-chlorophenyl)carbamoyl)phenoxy)acetate series; (**b**) ethyl 2-(2-((4-chlorophenyl) carbamoyl)phenoxy)acetate series.

FTIR is a useful tool to show the presence of host and guest molecules in the structure of inclusion complexes. FTIR spectra of the ethyl esters, β-cyclodextrin, physical mixtures, and inclusion complexes are presented in Figure 3.

The IR spectra of the inclusion complexes (CEE2, CEE4) looks almost similar to the one of pure β-CD, a phenomenon observed by other research that demonstrated the formation of the complex [27]. In addition, the broad band at 3396 cm^−1^, corresponding to the hydroxyl groups of pure cyclodextrin, appears sharper in the complex spectra, which is also proof of the complex’s formation, observed by other researchers [27,28,29]. On the other hand, the spectra of the physical mixture can be described as an overlap of the spectra of the pure compounds, being able to highlight in it both the characteristic bands of the ester and of the cyclodextrin.

Differences that occurred in the case of the ethyl esters in the IR spectra of the complexes with respect to the IR spectra of the free ethyl ester are presented in Table 1. These modifications show that the esters were entrapped with the salicylic part inside the CD cavity.

Some differences appear in the IR spectra of the inclusion complexes regarding the position of the absorption bands corresponding to CD (Table 2). Thus, the modified frequencies observed for β-cyclodextrin correspond to symmetric and asymmetric stretching vibrations ν[OH], ν[CH_2_], ν[C–C] and bending vibrations ν[O–H], respectively.

These changes in the IR spectra of the complexes show either decreases or increases in the frequency of certain bands. The increase is due to the inclusion of the benzene nucleus inside the cavity of β-cyclodextrin, which leads to the increase in electron cloud density [30]. The decreases in frequency are due to the alteration of the microclimate that leads to the development of hydrogen bonds and van der Waals attractions [31].

No new bands appeared in the inclusion complexes’ spectra, indicating that no chemical bonds were formed between the two components of the complexes.

**Figure 4:**
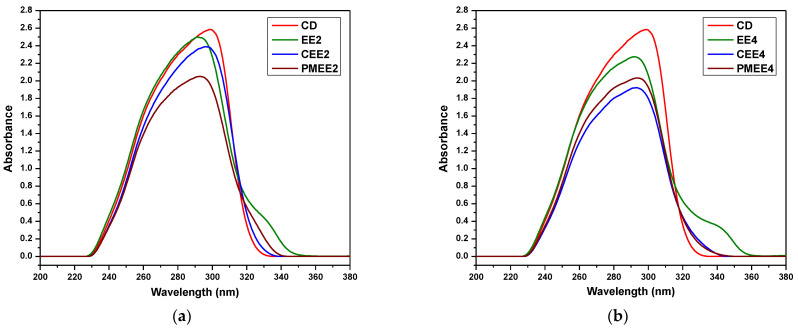
UV-vis spectra of β-cyclodextrin, ethyl ester, inclusion complex, physical mixture: (**a**) ethyl 2-(2-((2-chlorophenyl)carbamoyl)phenoxy)acetate series; (**b**) ethyl 2-(2-((4-chlorophenyl) carbamoyl)phenoxy)acetate series.

UV-vis behavior of the ethyl esters, β-cyclodextrin, physical mixtures, and inclusion complexes is presented in Figure 4.

UV-vis spectra show an absorption profile of the complexes (CEE2, CEE4) similar to the CD one. As for the physical mixtures (PME2, PME4), the profile is a combination of the two profiles of the parent compounds.

**Figure 5:**
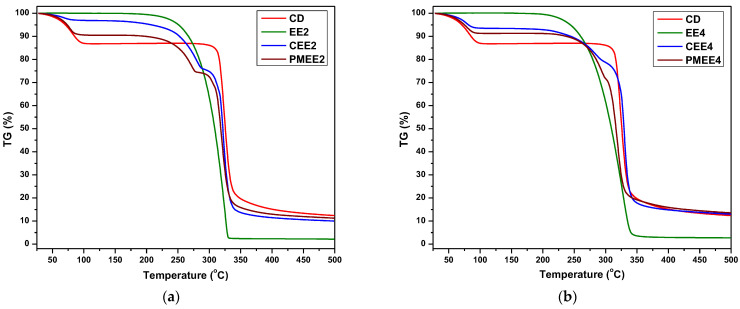
TGA curves of β-cyclodextrin, ethyl ester, inclusion complex, physical mixture: (**a**) ethyl 2-(2-((2-chlorophenyl)carbamoyl)phenoxy)acetate series; (**b**) ethyl 2-(2-((4-chlorophenyl) carbamoyl)phenoxy)acetate series.

Thermogravimetric analysis was performed to identify the mass loss in relation to the temperature change. Thermograms were recorded for pure β-cyclodextrin, ethyl esters, physical mixtures and for the inclusion complexes. The results obtained in the temperature range 20–500 °C are plotted in Figure 5.

β-Cyclodextrin show two mass losses. The first is due to the loss of water located in the CD cavity and appears as an inflection point in TG at 83.6 °C, and the second, the decomposition of the macrocycle, at 323.4 °C. The ethyl esters show a single loss of mass in the temperature range 200–340 °C, due to the degradation of benzene nuclei present in its structure. Regarding the behavior of the complexes, these present mass losses in three stages. The first stage corresponds to the loss of mass due to the dehydration of the molecules, the second stage is the decomposition of β-cyclodextrin, and the last one may be related to the decomposition of the ester. To confirm the formation of the inclusion complex, a thermal analysis of the physical mixture between β-cyclodextrin and ethyl esters was also performed. The first mass loss in the case of the inclusion complexes occurs at 70.4 °C (CEE2) and 78.3 °C (CEE4), while in the case of the physical mixtures, it occurs at 77.0 °C (PMEE2) and 80.1 °C (PMEE4) which are closer to the value corresponding to the loss of water in the case of pure CD. In addition, the second mass loss in the case of CD, corresponding to the degradation of the macrocycle, occurs at 323.4 °C, but in the case of the complexes, the values were higher (322.1 °C—CEE2; 329.1 °C—CEE4). All these phenomena suggest that the formation of the inclusion complexes changed the thermal stability of the cyclodextrin. This shows that the formation of the inclusion complexes altered the thermal degradation property of the two components as such.

Comparing the changes that occur in the X-ray spectra, SEM images, IR spectra, UV-vis spectra, and thermograms of the inclusion complexes with those recorded in the case of the pure cyclodextrin and ethyl esters, it can be concluded that using kneading, the predicted complexes were obtained.

### 3.4. Antibacterial Activity Evaluation

The antibacterial activity of the salicylanilide derivatives is shown in Table 3. It can be observed that only Gram-positive bacteria were susceptible to the tested compounds. Similar results regarding the lower susceptibility of Gram-negative bacteria to some esters of halogenated salicylanilide have been reported [6,7,32].

Krátký et al. (2010) [32] synthesized new amino acid esters of halogenated salicylanilide, (S)-2-(phenylcarbamoyl)phenyl 2-acetamido-3-phenylpropanoates and tested them against *Staphylococcus aureus*, methicillin-resistant *Staphylococcus aureus*, *Staphylococcus epidermidis*, *Enterococcus* spp., *Escherichia coli*, and *Klebsiella pneumoniae*. The most active compounds were (S)-2-(4-bromophenylcarbamoyl)-5-chlorophenyl 2-acetamido-3-phenylpropanoate and (S)-4-chloro-2-(4-(trifluoromethyl)phenylcarbamoyl)phenyl 2-acetamido-3-phenyl propanoate with MICs between 0.98 and 31.25 μmol/L. Gram-negative bacteria were less sensible (MICs = 15.62–500 μmol/L) [32].

Salicylanilide 4-(trifluoromethyl) benzoates were tested against *Staphylococcus aureus*, methicillin-resistant *Staphylococcus aureus*, *Staphylococcus epidermidis*, *Enterococcus* spp., and *E. coli*. For Gram-positive bacteria, the MICs were higher than 0.49 μmol/L, while *E. coli* was much less susceptible. The esterification improved slightly the cytotoxicity of the related salicylanilides containing unsubstituted OH group, which is possibly the principal factor responsible for their cytotoxicity (MICs ≥ 31.25 μmol/L). The growth of *P. aeruginosa* and *K. pneumoniae* was not reduced up to 500 μmol/L [6].

Other esters, salicylanilide diethyl phosphates, exhibited activity against Gram-positive bacteria with MICs higher than 1.95 µmol/L, Gram-negative species were resistant up to the concentration of 500 µmol/L [7].

In our study, compounds derived from N-(2-chlorophenyl)-2-hydroxybenzamide showed higher activity against Gram-positive bacteria strains, with MIC values ranging 0.125–0.5 mg/mL. Lower or no antibacterial effect was found with N-(4-chlorophenyl)-2-hydroxybenzamide series (MIC ≥ 0.5 mg/mL). The ethyl and methyl ester MICs were generally twice lower than in the case of hydrazides. Antibacterial activities of methyl esters were equal to those of ethyl esters, except EM4, which inhibited *S. mutans* and *S. pyogenes* more easily.

Among the chosen bacteria strains, the most susceptible to the action of chloro-substituted salicylanilide derivatives proved to be two of the major human-specific Gram-positive bacteria pathogens: *Streptococcus pyogenes*, which causes a wide collection of symptoms, from mild localized infections to invasive infections that can be associated with high morbidity and mortality [33], and *Streptococcus mutans*, which is related to pyogenic and other infections in the mouth, heart, joints, skin, muscle, and central nervous system [34].

In this research, the effect of cyclodextrin complexation on the antibacterial activity of the drug alone was studied for ethyl esters (EE2, EE4). We started from the premise that entrapping antimicrobial compounds in β-CD should lead to proper control of drug release, so that the drugs could be more efficiently used.

The β-CD complex of ethyl 2-(2-((2-chlorophenyl)carbamoyl)phenoxy)acetate (CEE2) preserved the same activity of the EE2 against the tested bacteria, even if the amount of the EE2 in the complex was approximately 4 times smaller. This mechanism of action can be related to the fact that complexation may disturb the cell membrane potential of bacteria and improve the membrane permeability of the drug. Similar findings were reported by Inoue et al. in a study that revealed that hinokitiol (HT), alone and HT/CD complexes, is active against *Bacillus subtilis*, *Staphylococcus aureus*, *Escherichia coli*, and *Pseudomonas aeruginosa*. No decrease in antimicrobial activity was observed, although HT was included in CD, and HT/CD complexes revealed equal or higher activity than that of HT alone [35].

As for the ethyl 2-(2-((4-chlorophenyl)carbamoyl)phenoxy)acetate complex (CEE4), low or lack of activity was observed. These findings can be attributed (1) to the significantly lower activity of the ethyl ester itself and (2) to the lower quantity of ester retrieved in the complex, and as such, in the tested concentrations as well (1 mg CEE4 contains only 0.22 mg EE4).

The MBC values were identical to MICs, so the tested compounds action is bactericidal against the sensitive bacteria.

## 4. Conclusions

In conclusion, we designed and obtained new chloro-substituted salicylanilide derivatives, esters, hydrazides, and ethyl ester inclusion complexes. The compounds were screened for their capacity to inhibit Gram-positive and Gram-negative bacterial strains, proving to have good bactericidal effect against the Gram-positive ones. 2-Chloro substitution of the aniline ring of salicylanilide seems to be beneficial for the antibacterial effect, so all of the tested compounds belonging to this series, but especially the esters, can be considered potential antibacterial agents. We have also demonstrated the possibility of using ethyl esters in complexed form to compete with bacteria, with all its advantages. Thus, these findings could be a valuable theme for research and development for clinical applications.

## Figures and Tables

**Table 1:** Modified characteristic vibrations of ethyl esters after complexation.

Characteristic Vibrations	EE2		EE4	
	Free (cm^−1^)	Complexed (cm^−1^)	Free (cm^−1^)	Complexed (cm^−1^)
Sk “1600”	1592.14	1593.11	1593.11	1596.97
γNH + νCN sec. amide	1535.25	1536.21	1533.32	1535.25
Sk “1500”	1484.14, 1438.81	1482.21, 1439.78	1436.88	1440.74
σCH_3_ aliphatic	1391.56	1394.45	1392.52	1396.38
σCH aromatic	1378.06	1375.17	1361.66	overlaped
νasCOC aliphatic	1161.08	1156.26	1157.22	1155.29

**Table 2:** Modified characteristic vibrations of β-cyclodextrin after complexation.

β-Cyclodextrin	Free (cm^−1^)	Complexed EE2 (cm^−1^)	Complexed EE4 (cm^−1^)
ν[OH]	3396.45	3395.48	3398.38
ν[CH_2_]	2925.84	2928.74	2925.84
ν[C–C]	1157.22	1156.26	1155.29
ν[O–H]	1028.96	1029.93	1033.79

**Table 3:** Antibacterial activity of test compounds.

Microbial Strains	Test Compounds	MIC (mg/mL, *)	MBC (mg/mL, *)
*S. mutans* ATCC 35668	EE2	0.25	0.25
	EM2	0.25	0.25
	HD2	0.5	0.5
	CEE2	0.25	0.25
		
	EE4	NA	NA
	EM4	0.5	0.5
	HD4	0.5	0.5
	CEE4	NA	NA
*S. pyogenes* ATCC 19615	EE2	0.125	0.125
	EM2	0.125	0.125
	HD2	0.25	0.5
	CEE2	0.125	0.125
		
	EE4	1.0	1.0
	EM4	0.5	0.5
	HD4	0.5	0.5
	CEE4	NA	NA
*S. aureus* ATCC 25923	EE2	0.5	0.5
	EM2	0.5	0.5
	HD2	1.0	1.0
	CEE2	0.5	0.5
		
	EE4	1.0	1.0
	EM4	1.0	1.0
	HD4	1.0	1.0
	CEE4	NA	NA
*E. coli* ATCC 25922	EE2	NA	NA
	EM2	NA	NA
	HD2	NA	NA
	CEE2	NA	NA
		
	EE4	NA	NA
	EM4	NA	NA
	HD4	NA	NA
	CEE4	NA	NA
*P. aeruginosa* ATCC 27853	EE2	NA	NA
	EM2	NA	NA
	HD2	NA	NA
	CEE2	NA	NA
		
	EE4	NA	NA
	EM4	NA	NA
	HD4	NA	NA
	CEE4	NA	NA

* NA—compounds exhibited no activity at tested concentrations.

## Data Availability

Not applicable.
